# Potassii Nitras in Chill and Fever

**Published:** 1890-02

**Authors:** J. D. Hunter

**Affiliations:** New Orleans, La.


For Daniel's Texas Medical Journal.

### POTASSII NITRAS IN CHILL AND FEVER.

BY J. D. HUNTER, M. D., NEW ORLEANS.

CHILL AND FEVER frequently resists the curative influences of quinine and arsenic, tends to become chronic and very protracted, leading to great physical exhaustion and incurable organic lesions.

I have discovered that potassii nitras is an unusually effective agent in the treatment of chill and fever. To speak summarily, I have, during the past five years, tested it most fully. At least sixty-five per cent. of all the cases tested have been cured by the administration of a single dose; 35 per cent. were uninfluenced by repeated doses. The best results were obtained when the drug was administered during the premonitory stage, which usually ushers in the chill. Twenty-five or thirty grains given at this period, will either abort the chill, or materially shorten its duration. The febrile stage is correspondingly shortened, or reduced to a minimum. A second dose is seldom required; relapse is infrequent. Recent attacks, as well as protracted conditions, were alike cured by the administration of a single dose, while cases apparently similar, corresponding in character and duration, were not relieved. Other forms of intermittent, not associated with chill, were not benefitted in a single instance.

In the employment of potassii nitras, I have kept the cases treated under close observation, and have observed the greatest accuracy in noting results. At my request, a few physicians in the country, where chill and fever prevails extensively, have employed the salt. Their experience was identical with mine as to the unusual rapidity and permanency of cures; the proportion of cures have, however, not equalled mine.

That a disorder extending over a protracted period of months or years, characterized by the regular occurrence of periodic malarial paroxysms, and presenting the characteristic evidences of chronic malarial poisoning, should be instantaneously cured by the administration of a comparatively infinitesimal quantity of potassii nitras, a rapid restoration to health following without subsequent treatment and without relapse, does not accord with our experience in the use of medicine, and may justly be held as new and unusual.

I have no theory to offer in explanation of the action of the salt. To attempt to render a reason would be a mere matter of doubtful speculation.

The clinical history and pathological results of the malarial poison have been profoundly studied and most fully elucidated, notably in the great work of Prof. Joseph Jones, M. D., of this city; but of the peculiar paroxysmal phenomena arising from the action of the malarial poison in the system, our knowledge is merely speculation; many elaborate theories have been offered, but they amount to little more than a plausible hypothesis.

Is the the malarial poison an organism? This theory is accepted as the most rational view, yet, though claimed to have been proven, it has not been fully and satisfactorily demonstrated to be a fact.

If the poison is an organism, are the manifestations in the system—chill and fever, intermittent, remittent, congestive, pernicious, etc., due to the intensity of the cause, degree and rapidity of development and reproduction, or to some modifying property in the system invaded, or is there a variety of organisms, each differing in effect and determining the special result? Scientific research has failed to throw any new light on the subject. Without positive demonstration, the whole matter, in the present state of our knowledge, may be considered as veiled in profound darkness.

In order to determine the exact value of potassii nitras in the treatment of chill and fever, I would request the profession to give it a full and careful trial, and favor us with the result of their experience, through this journal or by direct communication.

A large proportion of the morbid conditions of the system which we are called upon to treat, particularly in the Southern States, are of malarial origin, or are aggravated by a pre-existing malarial cachexia, consequently this subject, more than any other, should enlist the attention of every Southern physician. Prof. Joseph Jones, M. D., has given to the profession a work which, both in America and Europe, is held to be the most thorough and complete on this highly important subject. Every physician who practices in a malarial region should possess his volumes, and study them carefully.

---

THE "DIXIE DOCTOR" is the name of the latest addition to the medical journals of America. It is a neat two-column pamphlet of twenty pages of text, issued at Atlanta, Ga., edited by T. H. Huzza, M. D. Vol. I., No. I, is a neat little book.

